# 2,5-Dimethyl­anilinium chloride monohydrate

**DOI:** 10.1107/S1600536808041287

**Published:** 2008-12-10

**Authors:** Wajda Smirani, Mohamed Rzaigui

**Affiliations:** a Laboratoire de Chimie des Matériaux, Faculté des Sciences de Bizerte, 7021 Zarzouna, Bizerte, Tunisia

## Abstract

In the title compound, C_8_H_12_N^+^·Cl^−^·H_2_O, the crystal packing is influenced by O—H⋯Cl, N—H⋯Cl and N—H⋯O hydrogen bonds, resulting in a two-dimensional network propagating parallel to (001).

## Related literature

For related literature, see: Aloui *et al.* (2006 ▶); Masse *et al.* (1993 ▶); Blessing (1986 ▶). 
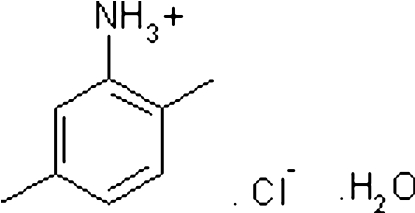


## Experimental

### 

#### Crystal data

C_8_H_12_N^+^·Cl^−^·H_2_O
*M* *_r_* = 175.65
Monoclinic, 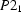

*a* = 7.515 (4) Å
*b* = 7.441 (3) Å
*c* = 9.019 (2) Å
β = 102.87 (3)°
*V* = 491.7 (4) Å^3^
*Z* = 2
Mo *K*α radiation
μ = 0.34 mm^−1^
*T* = 293 (2) K
0.50 × 0.30 × 0.20 mm

#### Data collection

Enraf–Nonius TurboCAD-4 diffractometer
Absorption correction: none
2058 measured reflections
1260 independent reflections
1166 reflections with *I* > 2σ(*I*)
*R*_int_ = 0.025
2 standard reflections frequency: 120 min intensity decay: 5%

#### Refinement

*R*[*F* ^2^ > 2σ(*F* ^2^)] = 0.028
*wR*(*F* ^2^) = 0.081
*S* = 1.10
1260 reflections
111 parameters
1 restraint
H atoms treated by a mixture of independent and constrained refinement
Δρ_max_ = 0.20 e Å^−3^
Δρ_min_ = −0.14 e Å^−3^
Absolute structure: Flack (1983 ▶), unique data only
Flack parameter: 0.17 (9)

### 

Data collection: *CAD-4 EXPRESS* (Enraf–Nonius, 1994 ▶); cell refinement: *CAD-4 EXPRESS*; data reduction: *XCAD4* (Harms & Wocadlo, 1995 ▶); program(s) used to solve structure: *SHELXS97* (Sheldrick, 2008 ▶); program(s) used to refine structure: *SHELXL97* (Sheldrick, 2008 ▶); molecular graphics: *ORTEP-3* (Farrugia, 1997 ▶); software used to prepare material for publication: *WinGX* publication routines (Farrugia, 1999 ▶).

## Supplementary Material

Crystal structure: contains datablocks I, global. DOI: 10.1107/S1600536808041287/hb2876sup1.cif

Structure factors: contains datablocks I. DOI: 10.1107/S1600536808041287/hb2876Isup2.hkl

Additional supplementary materials:  crystallographic information; 3D view; checkCIF report

## Figures and Tables

**Table 1:** Hydrogen-bond geometry (Å, °)

*D*—H⋯*A*	*D*—H	H⋯*A*	*D*⋯*A*	*D*—H⋯*A*
O1—H30⋯Cl1^i^	0.81 (4)	2.37 (4)	3.168 (3)	171 (4)
O1—H31⋯Cl1	0.78 (4)	2.44 (4)	3.219 (3)	174 (5)
N1—H1*A*⋯O1^ii^	0.89	1.82	2.705 (4)	171
N1—H1*B*⋯Cl1^iii^	0.89	2.29	3.167 (2)	169
N1—H1*C*⋯Cl1^iv^	0.89	2.30	3.189 (3)	173

Symmetry codes: (i) 
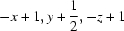
; (ii) 
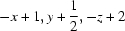
; (iii) 
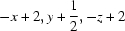
; (iv) 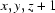.

**Fig. 1.**
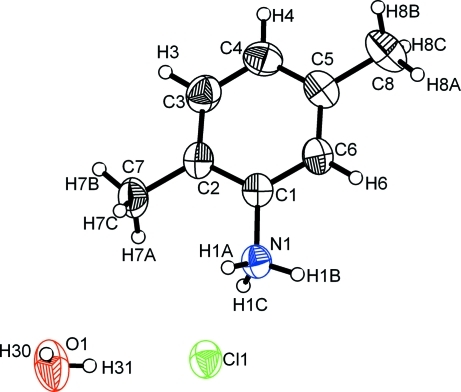
View of (I) with displacement ellipsoids for non-H atoms drawn at the 30% probability level.

**Fig. 2.**
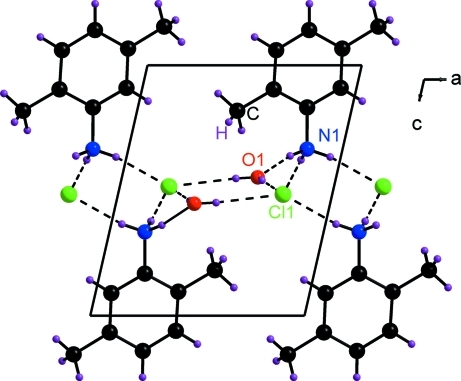
A view of the atomic arrangement of the title compound along the b axis with H bonds shown as dashed lines.

## supplementary crystallographic information

### Comment

The preparation of inorganic anion and organic cation salts continues to be a focus area in chemistry and material science because of their abilities to combine the properties of organic and inorganic compounds within one single molecular scale, so as to exhibit some interesting crystal structure and some special properties, such as second-order nonlinear optical response, magnetism, luminescence, and even multifunctional properties (Masse *et al.*, 1993). It is therefore vital to design and synthesize novel salts with inorganic anions and organic cations so as to explore their various properties. In this context, we report the synthesis and the crystal structure of a the title compound, (I), (Fig. 1). The crystal packing can be described as a typical layered organization. A projection of such a layer shows that the Cl^-^ anions are linked to the water molecules by O—H···Cl hydrogen bonds to form infinite corrugated chains along the b direction (Fig. 2). These chains are themselves connected *via* N—H···O and N—H···Cl hydrogen bonds originating from NH_3_^+ ^groups, so as to built inorganic layers spreading around the *(a,b) *plane. The 2,5-xylidinium cations are anchored onto the successive inorganic layers *via* hydrogen bonds and electrostatic interactions, to compensate their negative charges.

The examination of the organic cation shows that the values of the N—C, C—C distances and N—C—C, C—C—C angles range from 1.376 (3) to 1.503 (3) Å and 115.72 (19) to 122.80 (19)°, respectively. These values show no significant difference from those obtained in other organic materials associated with the same organic groups (Aloui *et al.*, 2006).

In this structure, the water molecule play a very important role in the cohesion of the various groups. It participates with the organic cation and chloride anion in an H-bonding scheme of N—H···O and O—H···Cl interactions in the asymmetrical unit. Among these five H-bonds, only one could be considered to be strong according to the well known criterion of Blessing and Brown: N···O = 2.705 (4)Å (Blessing, 1986). The four remaining hydrogen bonds are relatively weak, and their donor···acceptor distances vary from 3.167 (2) to 3.219 (3) Å. Thus, these different interactions (hydrogen bonds, Van der Waals, and electrostatic) form a stable three-dimensional network.

### Experimental

The title compound was prepared by slow addition, at room temperature, of an aqueous hydrochloric acid solution to an alcoholic solution of 2,5-xylidine in a 1:1 molar ratio. A crystalline precipitate was formed. After dissolution by adding H_2_O, the solution was slowly evaporated at room temperature over several days resulting in the formation of transparent plates of (I).

### Refinement

The water H atoms were located in a difference map and freely refined. The other H atoms were positioned geometrically (N—H = 0.89, C—H = 0.93–0.96 Å) and refined as riding with *U*_iso_(H) = 1.2U_eq_(C,N) or 1.5U_eq_(methyl C).

### Crystal data

**Table:**

C_8_H_12_N^+^·Cl^−^·H_2_O	*F*(000) = 188
*M**_r_* = 175.65	*D*_x_ = 1.187 Mg m^−^^3^
Monoclinic, *P*2_1_	Mo *K*α radiation, λ = 0.71073 Å
Hall symbol: P 2yb	Cell parameters from 25 reflections
*a* = 7.515 (4) Å	θ = 9.0–10.8°
*b* = 7.441 (3) Å	µ = 0.34 mm^−^^1^
*c* = 9.019 (2) Å	*T* = 293 K
β = 102.87 (3)°	Plate, colourless
*V* = 491.7 (4) Å^3^	0.50 × 0.30 × 0.20 mm
*Z* = 2	

### Data collection

**Table:**

Enraf–Nonius TurboCAD-4 diffractometer	*R*_int_ = 0.025
Radiation source: Enraf Nonius FR590	θ_max_ = 28.0°, θ_min_ = 2.3°
graphite	*h* = −9→9
non–profiled ω scans	*k* = 0→9
2058 measured reflections	*l* = −5→11
1260 independent reflections	2 standard reflections every 120 min
1166 reflections with *I* > 2σ(*I*)	intensity decay: 5%

### Refinement

**Table:**

Refinement on *F*^2^	Secondary atom site location: difference Fourier map
Least-squares matrix: full	Hydrogen site location: difmap and geom
*R*[*F*^2^ > 2σ(*F*^2^)] = 0.028	H atoms treated by a mixture of independent and constrained refinement
*wR*(*F*^2^) = 0.081	*w* = 1/[σ^2^(*F*_o_^2^) + (0.0515*P*)^2^ + 0.0061*P*] where *P* = (*F*_o_^2^ + 2*F*_c_^2^)/3
*S* = 1.10	(Δ/σ)_max_ < 0.001
1260 reflections	Δρ_max_ = 0.20 e Å^−^^3^
111 parameters	Δρ_min_ = −0.13 e Å^−^^3^
1 restraint	Absolute structure: Flack (1983), unique data only
Primary atom site location: structure-invariant direct methods	Flack parameter: 0.17 (9)

### Special details

**Table:**

Geometry. All e.s.d.'s (except the e.s.d. in the dihedral angle between two l.s. planes) are estimated using the full covariance matrix. The cell e.s.d.'s are taken into account individually in the estimation of e.s.d.'s in distances, angles and torsion angles; correlations between e.s.d.'s in cell parameters are only used when they are defined by crystal symmetry. An approximate (isotropic) treatment of cell e.s.d.'s is used for estimating e.s.d.'s involving l.s. planes.
Refinement. Refinement of *F*^2^ against ALL reflections. The weighted *R*-factor *wR* and goodness of fit *S* are based on *F*^2^, conventional *R*-factors *R* are based on *F*, with *F* set to zero for negative *F*^2^. The threshold expression of *F*^2^ > σ(*F*^2^) is used only for calculating *R*-factors(gt) *etc*. and is not relevant to the choice of reflections for refinement. *R*-factors based on *F*^2^ are statistically about twice as large as those based on *F*, and *R*- factors based on ALL data will be even larger.

### Fractional atomic coordinates and isotropic or equivalent isotropic displacement parameters (Å^2^)

**Table:**

	*x*	*y*	*z*	*U*_iso_*/*U*_eq_	
H30	0.343 (5)	0.410 (6)	0.541 (3)	0.068 (9)*	
H31	0.467 (5)	0.287 (7)	0.548 (4)	0.101 (13)*	
Cl1	0.77059 (6)	0.20498 (11)	0.51544 (5)	0.05039 (16)	
C1	0.7901 (2)	0.5395 (3)	1.1684 (2)	0.0384 (4)	
N1	0.8345 (2)	0.5589 (3)	1.33479 (17)	0.0417 (4)	
H1A	0.7624	0.6418	1.3617	0.062*	
H1B	0.9505	0.5923	1.3660	0.062*	
H1C	0.8174	0.4543	1.3774	0.062*	
C6	0.9259 (3)	0.5724 (3)	1.0904 (2)	0.0431 (4)	
H6	1.0405	0.6101	1.1431	0.052*	
C2	0.6147 (2)	0.4859 (3)	1.0962 (2)	0.0427 (4)	
C5	0.8914 (3)	0.5491 (3)	0.9335 (3)	0.0487 (5)	
C3	0.5844 (3)	0.4628 (4)	0.9399 (2)	0.0541 (5)	
H3	0.4698	0.4248	0.8873	0.065*	
C7	0.4674 (3)	0.4531 (4)	1.1817 (3)	0.0560 (6)	
H7A	0.5086	0.3654	1.2599	0.084*	
H7B	0.3597	0.4097	1.1128	0.084*	
H7C	0.4399	0.5634	1.2271	0.084*	
C4	0.7171 (3)	0.4937 (4)	0.8597 (3)	0.0551 (6)	
H4	0.6902	0.4773	0.7548	0.066*	
O1	0.3659 (3)	0.3043 (4)	0.5526 (3)	0.0783 (6)	
C8	1.0363 (4)	0.5817 (4)	0.8458 (3)	0.0682 (7)	
H8A	1.1339	0.6502	0.9071	0.102*	
H8B	0.9850	0.6472	0.7546	0.102*	
H8C	1.0828	0.4687	0.8197	0.102*	

### Atomic displacement parameters (Å^2^)

**Table:**

	*U*^11^	*U*^22^	*U*^33^	*U*^12^	*U*^13^	*U*^23^
Cl1	0.0441 (2)	0.0462 (2)	0.0622 (3)	0.0054 (3)	0.01468 (18)	0.0086 (3)
C1	0.0394 (9)	0.0301 (8)	0.0461 (9)	−0.0005 (7)	0.0105 (7)	−0.0040 (7)
N1	0.0388 (7)	0.0414 (8)	0.0458 (8)	−0.0036 (7)	0.0115 (6)	−0.0009 (7)
C6	0.0421 (9)	0.0341 (9)	0.0555 (11)	−0.0039 (8)	0.0157 (8)	−0.0023 (9)
C2	0.0378 (8)	0.0371 (9)	0.0541 (11)	−0.0006 (8)	0.0120 (8)	−0.0042 (9)
C5	0.0574 (11)	0.0372 (9)	0.0567 (11)	−0.0024 (9)	0.0237 (9)	−0.0033 (10)
C3	0.0486 (11)	0.0568 (14)	0.0545 (12)	−0.0063 (11)	0.0064 (9)	−0.0123 (11)
C7	0.0400 (10)	0.0655 (16)	0.0649 (14)	−0.0104 (11)	0.0169 (9)	−0.0068 (12)
C4	0.0658 (13)	0.0554 (13)	0.0450 (11)	−0.0011 (12)	0.0143 (9)	−0.0082 (10)
O1	0.0586 (11)	0.0618 (13)	0.1229 (18)	0.0097 (10)	0.0381 (11)	0.0362 (12)
C8	0.0852 (18)	0.0626 (17)	0.0694 (15)	−0.0123 (15)	0.0439 (14)	−0.0039 (14)

### Geometric parameters (Å, °)

**Table:**

C1—C6	1.383 (3)	C3—C4	1.376 (3)
C1—C2	1.393 (3)	C3—H3	0.9300
C1—N1	1.470 (2)	C7—H7A	0.9600
N1—H1A	0.8900	C7—H7B	0.9600
N1—H1B	0.8900	C7—H7C	0.9600
N1—H1C	0.8900	C4—H4	0.9300
C6—C5	1.391 (3)	O1—H30	0.80 (4)
C6—H6	0.9300	O1—H31	0.78 (4)
C2—C3	1.388 (3)	C8—H8A	0.9600
C2—C7	1.503 (3)	C8—H8B	0.9600
C5—C4	1.393 (3)	C8—H8C	0.9600
C5—C8	1.501 (3)		
			
C6—C1—C2	122.80 (19)	C4—C3—H3	118.7
C6—C1—N1	118.46 (17)	C2—C3—H3	118.7
C2—C1—N1	118.73 (18)	C2—C7—H7A	109.5
C1—N1—H1A	109.5	C2—C7—H7B	109.5
C1—N1—H1B	109.5	H7A—C7—H7B	109.5
H1A—N1—H1B	109.5	C2—C7—H7C	109.5
C1—N1—H1C	109.5	H7A—C7—H7C	109.5
H1A—N1—H1C	109.5	H7B—C7—H7C	109.5
H1B—N1—H1C	109.5	C3—C4—C5	120.8 (2)
C1—C6—C5	120.3 (2)	C3—C4—H4	119.6
C1—C6—H6	119.9	C5—C4—H4	119.6
C5—C6—H6	119.9	H30—O1—H31	110 (4)
C3—C2—C1	115.72 (19)	C5—C8—H8A	109.5
C3—C2—C7	121.90 (19)	C5—C8—H8B	109.5
C1—C2—C7	122.38 (19)	H8A—C8—H8B	109.5
C6—C5—C4	117.7 (2)	C5—C8—H8C	109.5
C6—C5—C8	121.6 (2)	H8A—C8—H8C	109.5
C4—C5—C8	120.7 (2)	H8B—C8—H8C	109.5
C4—C3—C2	122.7 (2)		
			
C2—C1—C6—C5	−1.2 (3)	C1—C6—C5—C8	−179.4 (2)
N1—C1—C6—C5	177.61 (19)	C1—C2—C3—C4	−1.2 (4)
C6—C1—C2—C3	1.5 (3)	C7—C2—C3—C4	179.4 (3)
N1—C1—C2—C3	−177.3 (2)	C2—C3—C4—C5	0.6 (4)
C6—C1—C2—C7	−179.1 (2)	C6—C5—C4—C3	−0.2 (4)
N1—C1—C2—C7	2.1 (3)	C8—C5—C4—C3	179.7 (3)
C1—C6—C5—C4	0.5 (3)		

### Hydrogen-bond geometry (Å, °)

**Table:**

*D*—H···*A*	*D*—H	H···*A*	*D*···*A*	*D*—H···*A*
O1—H30···Cl1^i^	0.81 (4)	2.37 (4)	3.168 (3)	171 (4)
O1—H31···Cl1	0.78 (4)	2.44 (4)	3.219 (3)	174 (5)
N1—H1A···O1^ii^	0.89	1.82	2.705 (4)	171
N1—H1B···Cl1^iii^	0.89	2.29	3.167 (2)	169
N1—H1C···Cl1^iv^	0.89	2.30	3.189 (3)	173

Symmetry codes: (i) −*x*+1, *y*+1/2, −*z*+1; (ii) −*x*+1, *y*+1/2, −*z*+2; (iii) −*x*+2, *y*+1/2, −*z*+2; (iv) *x*, *y*, *z*+1.
